# Not Only *RET* but *NF1* and Chromosomal Instability Are Seen in Young Patients with Sporadic Medullary Thyroid Carcinoma

**DOI:** 10.1210/jendso/bvae059

**Published:** 2024-03-30

**Authors:** Luciana Audi Castroneves, Flavia Regina Rotea Mangone, Antonio Marcondes Lerario, Ana Maria da Cunha Mercante, Rafael Loch Batista, Luciana Rodrigues Carvalho Barros, Carla Vaz Ferreira, Evelin Cavalcante Farias, Felipe Augusto Brasileiro Vanderlei, Ana Luiza Maia, Maria Aparecida Nagai, Alexander Augusto Lima Jorge, Ana Oliveira Hoff

**Affiliations:** Endocrinology, Instituto do Câncer do Estado de São Paulo, São Paulo 01252-000, Brazil; Laboratory of Molecular Genetics, Center for Translational Research in Oncology (LIM-24), Instituto do Câncer do Estado de São Paulo, São Paulo 01252-000, Brazil; Endocrine Oncology, University of Michigan Ann Arbor 48109, USA; Pathology, Instituto do Câncer do Estado de São Paulo, São Paulo 01252-000, Brazil; Endocrinology, Instituto do Câncer do Estado de São Paulo, São Paulo 01252-000, Brazil; Laboratory of Molecular Genetics, Center for Translational Research in Oncology (LIM-24), Instituto do Câncer do Estado de São Paulo, São Paulo 01252-000, Brazil; Thyroid Unit, Endocrine Division, Hospital de Clínicas de Porto Alegre, Porto Alegre 90035-903, Brazil; Endocrinology, Instituto do Câncer do Estado de São Paulo, São Paulo 01252-000, Brazil; Head and Neck Surgery, Hospital das Clínicas da Faculdade de Medicina da USP, São Paulo 05403-010, Brazil; Thyroid Unit, Endocrine Division, Hospital de Clínicas de Porto Alegre, Porto Alegre 90035-903, Brazil; Laboratory of Molecular Genetics, Center for Translational Research in Oncology (LIM-24), Instituto do Câncer do Estado de São Paulo, São Paulo 01252-000, Brazil; Genetic Endocrinology Unit, Cellular and Molecular Endocrinology Laboratory (LIM-25) Faculdade de Medicina da Universidade de São Paulo, São Paulo 01246-903, Brazil; Endocrinology, Instituto do Câncer do Estado de São Paulo, São Paulo 01252-000, Brazil

**Keywords:** medullary thyroid carcinoma, exome, chromosomal instability, *NF1*, *RET*

## Abstract

**Context:**

Genetic analysis of sporadic medullary thyroid carcinoma (MTC) has revealed somatic variants in *RET*, *RAS*, and occasionally other genes. However, around 20% of patients with sporadic MTC lack a known genetic driver.

**Objective:**

To uncover potential new somatic or germline drivers, we analyze a distinct cohort of patients with sporadic, very early–onset, and aggressive MTC.

**Methods:**

Germline and somatic DNA exome sequencing was performed in 19 patients, previously tested negative for germline *RET* variants.

**Results:**

Exome sequencing of 19 germline samples confirmed the absence of *RET* and identified an *NF1* pathogenic variant in 1 patient. Somatic sequencing was successful in 15 tumors revealing *RET* variants in 80%, predominantly p.Met918Thr, which was associated with disease aggressiveness. In *RET*-negative tumors, pathogenic variants were found in *HRAS* and *NF1*. The *NF1* germline and somatic variants were observed in a patient without a prior clinical diagnosis of neurofibromatosis type 1, demonstrating that the loss of heterozygosity of *NF1* functions as a potential MTC driver. Somatic copy number alterations analysis revealed chromosomal alterations in 53.3% of tumors, predominantly in *RET*-positive cases, with losses in chromosomes 9 and 22 being the most prevalent.

**Conclusion:**

This study reveals that within a cohort of early-onset nonhereditary MTC, *RET* remains the major driver gene. In *RET*-negative tumors, *NF1* and *RAS* are drivers of sporadic MTC. In addition, in young patients without a *RET* germline mutation, a careful clinical evaluation with a consideration of germline *NF1* gene analysis is ideal to exclude Neurofibromatosis type 1 (NF1).

Medullary thyroid carcinoma (MTC) occurs in 2 primary forms: sporadic (75-80%) and hereditary (20-25%) [1-4]. Hereditary MTC is linked to multiple endocrine neoplasia type 2 (MEN2), an autosomal dominant syndrome categorized as MEN2A or MEN2B based on clinical traits. MEN2A involves MTC, pheochromocytoma, and hyperparathyroidism. Conversely, MEN2B, the rarer form (5%), has a notably early-onset, aggressive MTC, pheochromocytoma, mucosal neuromas, and marfanoid phenotype. The hallmark of hereditary MTC is the existence of a germline *RET* proto-oncogene pathogenic variant [5, 6].

Sporadic MTC typically presents in the fourth to sixth decades, with somatic *RET* variants as prevalent oncogenes (40-50% in unselected cohorts, up to 85% in advanced cases) [5, 7-12]. Among the *RET* variants, p.Met918Thr stands out as the most prevalent and is associated with an aggressive disease course and unfavorable prognosis [10, 11, 13-17]. Less frequent *RET* variants encompass missense variants at codons 611, 618, 620, 630, 632, 634, 768, 791, and 883, in addition to small indels. *HRAS* and *KRAS* variants account for 20% to 30% of the sporadic MTC cases [7, 8, 15, 18-21] and recently, was reported 3 cases of sporadic MTC devoid of *RET* and *RAS* alterations, but instead marked by biallelic inactivation of *NF1*, leading to loss of heterozygosity (LOH) in a tumor suppressor gene known to activate the Ras pathway signaling [22, 23]. The COSMIC (Catalogue of Somatic Mutations in Cancer) database indicates a substantial proportion (around 30%) of sporadic MTC cases lacking unknown drivers, and current research has narrowed it to less than 20% [7, 22-24].

This study explores the molecular profile of a selected cohort of patients with MTC with early-onset and aggressive disease without a pathogenic *RET* germline variant. This subgroup of patients caught our attention due to the early age of presentation, lack of a germline *RET* variant and high frequency of local and distant metastatic disease (94% and 68.4%, respectively), similar to our cohort of patients with MEN2B (100% and 72.2%; *P* = .48 and *P* = .85, respectively). The structural progression of disease with systemic treatment requirements was also similar between these 2 group of patients.

## Material and Methods

### Patients

From a cohort of 270 patients with MTC, including 101 sporadic cases, we selected 19 individuals with sporadic MTC, and advanced and early-onset disease. We defined early onset of disease as a diagnosis before the age of 30, considering that sporadic MTC typically manifests between the fourth and sixth decades of life [5]. We conducted whole exome-sequencing on primary or metastatic tumors when the primary tumor was not available, along with matched DNA extracted from blood samples.

The study, approved by the local ethics committee (NP1164/17, approval granted on October 2nd, 2017), required all participants to provide written informed consent after receiving a full explanation of the purpose and nature of all procedures.

### Methods

Clinical data were obtained from medical records. Genomic DNA was extracted from the patient's blood lymphocytes, while tumor DNA was extracted from formalin-fixed paraffin-embedded or fresh frozen tumors. An experienced pathologist selected tumoral areas with over 90% tumor cells. For 3 tumors with lower tumor content (around 70%), manual macrodissection was performed to increase the tumor percentage. Germline DNA was extracted by the QIAamp DNA Blood Midi kit (Qiagen#51185), and tumor DNA extraction employed the QIAamp DNA FFPE Advanced UNG kit (Qiagen#56704) and AllPrep DNA/RNA kit (Qiagen#80204) for frozen tumors. DNA quality was analyzed with the Infinium HD FFPE QC Assay kit (Illumina#WG-321-1001).

### Whole Exome Sequencing and Analysis

Peripheral blood DNA and tumor genomic DNA samples were randomly fragmented into 300 to 400 bp fragments with adapters attached at both ends using bead-linked transposomes. Library preparation followed Illumina's DNA Prep with Enrichment guidelines (Illumina#20025523). Library quantification and quality analysis were conducted with Qubit Broad Range kit and Tape Hight Sensitivity kit (Agilent#5067-5585), respectively.

Each captured library was loaded onto the HiSeq 2500 platform with HiSeqSBS kit V4 150 cycles (Illumina). Adapter sequences were removed from raw data using the bbduk tool from bbmap, and raw data underwent quality control analysis with fastqc. Paired-end reads aligned to the hs37d5 (hg19 + decoy) assembly of the human genome using minimap2 (version2.24-r1122). Reads sorting and duplicate marking were performed with the bamsormadup tool from biobambam2 (version2.0.183).

Germline variants were called using freebayes, while somatic variants were identified with Mutect2 from GATK (version 4.2.6.0) following recommended best practices workflows. Germline and somatic variants were annotated with Annovar, Franklin by Genoox, and VEP (Ensembl Variant Effect Predictor). All germline variants were classified according to the American College of Medical Genetics and Genomics guidelines, we included all variants classified as pathogenic or likely pathogenic [25]

Mutalyzer was applied to confirm variant nomenclature [26] Somatic copy number alterations (SCNAs), LOH analysis, and estimation of tumor ploidy and purity were performed using PureCN.

### Statistical Analysis

Categorical variables were analyzed by chi-squared and Fisher's tests when appropriate, followed by Cramer's V test when significant. The Kruskal‒Wallis test was used for the comparison of primary tumor size and the categorical variables listed above. *P* < .05 was considered to be statistically significant.

## Results

### Clinical Characteristics of the Patients

Among the 19 selected patients, the median age was 21 years (range 8-29). Four out of 19 patients (21%) were 12 years of age or younger and an additional 4 out of 19 (21%) patients were between the ages of 13 and 18. Additionally, 14 out of 19 patients (73.7%) were female. All had cervical lymph node metastases at diagnosis, with 10.5% (2/19) having distant metastases. The median primary tumor size was 4 cm (range 0.6-10.3), multifocality was detected in 26.3% (5/19), and C-cell hyperplasia areas were present in 10.5% (2/19). Median follow-up duration was 9.8 years (range 0.5-29), and, at the time of analysis, 73.7% (14/19) had distant metastases, with 47% (9/19) having 3 or more metastatic sites. Bone metastases were the most common (78.5%), followed by lung and liver (64.3%). During follow-up, 10 patients (52.6%) experienced radiographic disease progression requiring tyrosine kinase inhibitor (TKI) therapy. Two patients died due to disease progression at the ages of 17 and 30. Clinical and follow-up characteristics are summarized (Tables 1 and 2).

**Table 1.** Patients characteristics of the study cohort

Patient characteristics	No. of patients (%)
Number of patients	19
Female sex	14 (73.7%)
Median age at diagnosis (range), years	21 (8-29)
*RET* germline mutation	0
Median size of primary tumor (cm)	4 (0.6-10.3)
Multifocality/C-cell hyperplasia	5 (26%)/2 (10.5%)
Initial tumor stage (AJCC 8)	
I	0
II	0
III	4 (21)
IVA	11 (57.8)
IVB	0
IVC	3 (15.7)
Initial treatment	
Thyroidectomy and central LN dissection	17 (89.4)
+ Lateral LN dissection	10 (52.6)
Unresectable	2 (10.5)
Response to initial treatment	
Excellent	0
Biochemical incomplete	2 (10.5)
Structural incomplete	17 (89.4)

Abbreviations: LN, lymph node; *RET*, rearranged during transfection gene;

**Table 2.** Follow-up and therapeutic characteristics of the study cohort

Follow-up characteristics	(%)
Median calcitonin of patients not requiring TKI*^a^* (range), pg/mL	647 (12.5-4477)
Median calcitonin of patients requiring TKI*^a^*	11 776 (125-139 165)
Calcitonin doubling time before TKI	
<12 months	2 (20)
>24 months	3 (30)
Unknown*^b^*	5 (50)
Median CEA of patients not requiring TKI (range), ng/mL	2.9 (1.1-114)
Median CEA of patients requiring TKI*^a^* (range), ng/mL	468 (19.9-6894)
CEA doubling time before TKI	
<12 months	1 (10)
>24 months	4 (40)
Unknown*^b^*	5 (50)
Median follow-up (range), years	9.8 (0.5-25)
Distant metastases at the end of follow-up	14/19 (73.7)
Sites of metastases at the end of follow-up	
Liver	9/14 (64.3)
Mediastinal lymph nodes	5/14 (35.7)
Lung	9/14 (64.3)
Bones	11/14 (78.5)
Systemic treatment (TKI)	11/19 (57.8)
First-line vandetanib	7/11 (63.6)
First-line cabozantinib	2/11 (18.2)
First-line sorafenib	2/11 (18.2)
Second-line vandetanib	1/11 (9)
Median age at initiation TKI (years)	22 (11-43)
Median duration of treatment (range), months	56 (1.5-144)
Median duration of follow up (range), months	122 (7-346)
Disease related deaths	2/19 (10.5)

Abbreviations: CEA, carcinoembryonic antigen; TKI, tyrosine kinase inhibitor.

^*a*^At initiation of TKI.

^*b*^Two patients initiated therapy at diagnosis.

### Spectrum of the Germline Analysis

**Figure 1.**
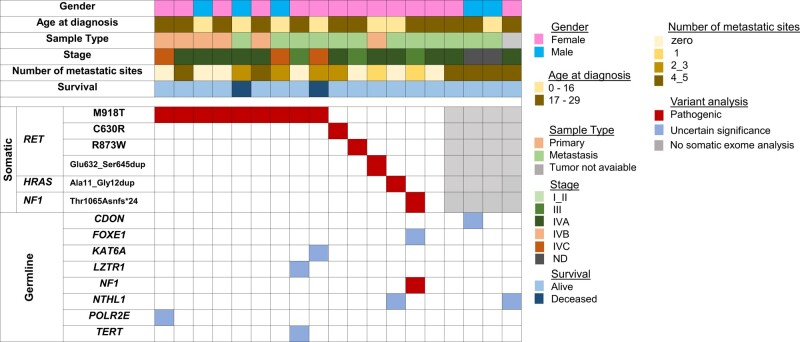
Mutational profile of the 19 sporadic MTC cases diagnosed before 30 years old, identified by the somatic (top) and germline (bottom) exome. Each column corresponds to a single case. Genetic variations are listed on the left. The red squares correspond to pathogenic variants, whereas the blue squares correspond to variants of uncertain significance (VUS) for the MTC phenotype and the gray squares identify 4 cases without somatic exome analysis: tumor not available (1 case) and bad quality DNA (3 cases).

Exome sequencing of 19 germline specimens revealed a patient harboring an *NF1* variant (c.1527 + 1G>T), classified as pathogenic by American College of Medical Genetics and Genomics, not found in population databases (ExAC), and associated with NF1 (Fig. 1) [27, 28]. Experimental studies confirmed its impact on mRNA splicing and protein function disruption [27].

No germline *RET* variants were identified in the cohort, and aside from the *NF1* variant, no other pathogenic variants were identified. Variants of unknown significance (VUS) of genes involved in tumorigenesis such as *FOXE1*, *KAT6A*, *LZTR1*, *NTHL1*, *POLR2E*, and *TERT* were observed in 5 patients harboring somatic drivers. Additionally, VUS (*CDON* and *NTHL1*) were identified in 2 patients for whom tumor DNA was not available.

### Spectrum of the Somatic Analysis—Single Variants

Exome somatic sequencing was performed on 18 available tumors: 3 were excluded due to poor DNA quality, leaving us with 15 tumors. DNA was obtained from the primary thyroid tumor in 40% (6/15) and lymph node metastases in 60% (9/15) of cases. Most samples (93.3%, 14/15) were formalin-fixed paraffin-embedded, with 1 fresh frozen tumor from a lymph node biopsy.

In our cohort, 93.3% (14/15) harbored single mutually exclusive somatic alterations, with 1 case showing no variants. *RET* was the most frequently altered gene, found in 80% (12/15). The most common *RET* somatic variant was p.Met918Thr in exon 16, present in 60% (9/15). Other *RET* variants were observed in 20% (3/15) of patients, including 2 reported in the COSMIC database (p.Cys630Arg and p.Arg873Trp) [24], and 1 novel *RET* in-frame insertion, p.Glu632_Ser645dup (Table 3).

**Table 3.** Somatic pathogenic variants identified in 19 young patients with sporadic medullary thyroid carcinoma

Pt	Tissue	Tumor cells %	Ploidy	Driver	Pathogenic variant	AF	Chromosomal changes
1	LN	71	1.911	*RET*	p.C630R	0.375	3/22 losses
2	Thyroid	63	1.991	*RET*	p.M918T	0.332	22 loss
3	LN	28	1.962	*NF1*	p.Thr1065Asnfs*24	0.284	—
4	LN	59	1.971	*RET*	p.R873W	0.33	11q/21 losses
5	Thyroid	59	1.922	*RET*	p.M918T	0.289	8p/9q loss
6	LN	70	1.929	*HRAS*	p.Ala11_Gly12dup	0.392	1p/4q losses
7	Thyroid	70	1.990	*RET*	p.M918T	0.2	—
8	Thyroid	59	1.987	*RET*	p.M918T	0.213	9 copy neutral LOH
9	LN	59	2.007	*RET*	p.M918T	0.212	—
11	Thyroid	84	1.955	*RET*	p.M918T	0.454	—
12	Thyroid	55	1.977	*RET*	p.Glu632_Ser645dup	0.513	19 p copy gain 21/22 losses
13	LN	55	1.980	*—*	—	—	—
14	LN	83	1.987	*RET*	p.M918T	0.28	—
15	LN	55	2.019	*RET*	p.M918T	0.249	—
19	LN	64	1.700	*RET*	p.M918T	0.334	1p/3/4/5q/9/13 losses

Abbreviations: AF, allele fraction; *HRAS*, HRas proto-oncogene gene; LOH, loss of heterozygosity; LN, lymph node; *NF1*, neurofibromin1 gene; Pt, patient number; *RET*, rearranged during transfection gene.

Only 1 *HRAS* pathogenic variant was detected (6.6%), an in-frame insertion variant (p.Ala11_Gly12dup) with a known pathogenic association with MTC (Table 3) [20]. This patient was diagnosed with MTC at age 8 and showed long-term follow-up with stable biochemical disease (calcitonin 12.5 pg/mL, carcinoembryonic antigen [CEA] 1.93 ng/mL) over 15.6 years.

A novel somatic *NF1* in-frame insertion variant (p.Thr1065Asnfs*24) was found in the same patient with a germline *NF1* variant (c.1527 + 1G>T), suggesting biallelic inactivation of this tumor suppressor gene (Table 3). Interestingly, this 22-year-old woman was referred with suspicion of MEN2B due to the presence of a marfanoid habitus but without other MEN2B features. Evaluation failed to reveal a *RET* germline variant but the finding of 3 small café-au-lait spots suggested an undefined genetic syndrome. Upon finding the germline *NF1* variant, several years after presentation, a more detailed examination revealed 12 café-au-lait spots and axillary freckling but no neurofibromas. At diagnosis, preoperative serum calcitonin was 3182 pg/mL. Total thyroidectomy with bilateral central node dissection revealed a 2-cm unifocal MTC with C-cell hyperplasia and central neck lymph nodes metastases (T1bN1a). She remained with biochemical incomplete disease until 3 years later when cervical recurrence was documented and treated with neck dissection. Seven years after diagnosis, liver metastases were detected but disease remains stable and under surveillance.

### Spectrum of the Somatic Analysis—SCNA Analysis

Tumor chromosomal instability, assessed via SCNA analysis (involving whole chromosome or single arm alterations), affected 53.3% (8/15) of cases, as detailed in Table 3 and elsewhere (Figs. S1-S8) [29].

Analyzing the SCNA prevalence based on somatic *RET* variants, among the 12 *RET*-positive tumors, 7 (58.3%) exhibited SCNAs, as did 1 of 3 *RET*-negative tumors. The tumor harboring the *NF1* variant did not show any SCNA. In the *RAS*-positive tumor, 2 partial losses occurred in chromosomes 1 and 4. Within the 7 *RET*-positive tumors with SCNA, there were 17 chromosome losses, 1 chromosome gain, and 1 copy-neutral LOH event. The affected chromosome count varied: 1 in 2 cases, 2 in 4 cases, and 1 with multiple monosomies (chromosomes 1p, 3, 4, 5q, 9, and 13). The most common losses were in chromosomes 9 and 22, each present in 37.5% (3/8) of SCNA-positive tumors, exclusively in *RET*-positive cases.

### Correlation of the Somatic Findings With Prognosis

We have correlated the presence of somatic *RET* and or SCNA with several prognostic features: TNM at diagnosis, development of distant metastasis during follow-up, need for systemic treatment, or mortality. Within this cohort of young patients and aggressive disease, we could still find a significant correlation between the presence of a *RET* variant and the need for systemic treatment (*P* = .03; *V* = 0.53). However, we could not find a significant correlation between the number of SCNAs per tumor, or the presence of losses in chromosomes 22 and 9 with worse outcomes, independent of the presence of a *RET* variant (*P* = .77).

**Figure 2.**
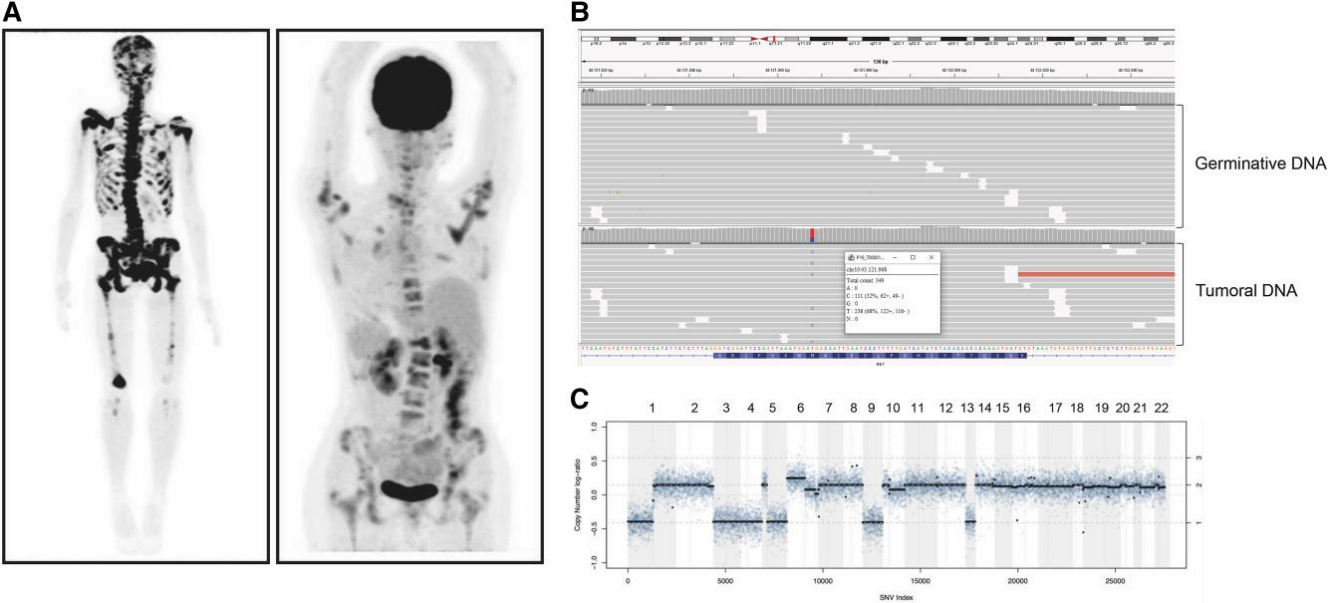
Clinical and tumor molecular characteristics of the patient harboring multiple somatic monosomy. (A) Bone scan and ^18^F-FDG PET/CT showing multiple lytic lesions involving the skull, and axial and appendicular skeleton. (B) Result of NGS showing absence of *RET* germinative mutation and presence of a somatic M918T mutation. (C) Somatic copy number alteration analysis (SCNA) with losses of chromosomes 1p, 3, 4, 5, 9, and 13.

Despite not observing a significant correlation between SCNA and poor prognosis in our cohort, 1 case presenting with multiple chromosome losses (1p/3/4/5q/9 and 13) drew our attention. A 16-year-old patient (patient 19, Table 3) with a *RET* 918 MTC presented with locally advanced disease and painful extensive bone involvement with disproportionately elevated CEA to calcitonin levels, 79 pg/mL and 4999 ng/mL, respectively; suggesting disease dedifferentiation. Despite receiving bone radiotherapy, cryoablation, and vandetanib, she continued to progress and died within 6 months (Fig. 2).

### Clinical and Pathological Characteristics of the Patients Harboring the SCNA in the Chromosome 22

Three patients harboring single *RET* somatic variants (p.Cys630Arg, p.Met918Thr and p.Glu632_Ser645dup) showed complete loss of chromosome 22. Two were diagnosed with MTC at 18 and 29 years old, presenting metastatic neck lateral lymph node disease. After 4 and 15 years, they initiated sorafenib treatment (a RET and RAF kinase and vascular endothelial growth factor inhibitor) due to progressive metastases involving more than 3 sites. Remarkably, they have demonstrated enduring responses, surpassing 10 years on treatment. The third patient, diagnosed with MTC (T2N1a) at age 15, developed stable metastatic liver disease after 3 years and has remained stable to date.

## Discussion

In our pursuit for a deeper understanding of sporadic MTC pathogenesis, we assembled an enriched cohort, predominantly composed of young patients with nonhereditary MTC. These individuals were selected from a larger cohort of 270 patients with MTC, of whom 101 had sporadic disease. Within this subset of sporadic cases, 19 patients were chosen based on their diagnosis before the age of 30, and, among them, 8 received their diagnoses before reaching the age of 18. All of these patients exhibited aggressive clinical profiles similar to our patients with MEN2B. This cohort stands as a unique population in whom we believed the identification of distinct genetic variants would be more likely.

In this study, we conducted germline and somatic exome analyses on 19 and 15 patients, respectively. Germline testing confirmed the absence of a germline *RET* variant and revealed an *NF1* pathogenic variant (c.1527 + 1G>T). No other germline pathogenic variants were identified.

Among the 15 patients with tumor profiling, 93.3% (14/15) harbored single and mutually exclusive somatic alterations. In 1 case, where both blood and tumor samples were analyzed, an extensive analysis failed to reveal a clear driver. Regarding the somatic pathogenic variants well-known to cause MTC, *HRAS* occurred in 6.6% (1/15), whereas *RET* variants were present in 80% (12/15). Notably, in a patient without somatic *RET* and *RAS*, both germline and somatic *NF1* pathogenic variants were identified.

Regarding the 12 patients harboring *RET* somatic variants, p.Met918Thr was the most common in 75% (9/12) and other *RET* somatic variants (p.Cys630Arg, p.Arg873Trp and 1 novel insertion in-frame p.Glu632_Ser645dup) were found in 25% (3/12) of patients. In the literature, sporadic MTC has somatic *RET* variants as the most prevalent driver oncogene involved in tumorigenesis, reaching up to 85% in advanced cases [10, 17]. Clearly, the most frequent *RET* somatic variant is p.Met918Thr [10, 11, 13, 15, 16] which exhibits higher frequency in studies that included patients with primary tumor size exceeding 2 cm and advanced disease [13]. We have validated this findings within our cohort, solidifying *RET* as the primary driver oncogene, even in young patients with aggressive disease, as observed in 80% of our cases. Notably, the p.Met918Thr pathogenic variant was the most common, accounting for 75% of our *RET* cases. Within this cohort, we were able to demonstrate that the presence of any somatic *RET* variant significantly correlated with disease progression requiring systemic treatment, as previously observed in nonselected MTC cohorts [10, 11].

Regarding SCNAs, we could not find significant correlation between the number of SCNAs per tumor or the presence of chromosome 22 and 9 losses with a worse outcome, independent of the presence of a *RET* variant (*P* = .77). In our cohort, an SCNA was observed in 53.3% (8/15) of tumors. The most prevalent were losses of chromosomes 9 and 22, occurring only in *RET*-positive tumors, as reported previously [30]. Association with poor outcomes has been previously observed with alterations in chromosomes 3, 9, 10, and 16, but not with SCNAs in chromosome 22 [30, 31].

We have not found a correlation between chromosome 9 alterations and poor prognosis, likely because our cohort was predominantly composed of patients with aggressive disease. However, in our patient with the most aggressive disease, tumor analysis revealed a complete loss of chromosome 9 in addition to multiple other losses (1p, 3, 4, 5q and 13). She presented with poorly differentiated MTC at age 16 and failed local and systemic treatments, rapidly progressing to death (Fig. 2, Table 3).

In the study by Ramone et al, chromosome 22 loss did not correlate with poor outcome which agrees with our findings [30]. Chromosome 22 encompasses several tumors suppressor genes such as *LZTR1*, *NF2*, *CHEK2*, and *SMARCB1*. In fact, pathogenic variants leading to the inactivation of the *LZTR1* have been linked to strong activation of the Ras/MAPK pathway leading to diseases such as schwannomatosis and RASopathies [32]. Interestingly, in our cohort, 2 out of 3 patients exhibiting chromosome 22 loss have demonstrated a sustained response to sorafenib, a multikinase inhibitor with potent inhibitory effects on Raf-1 and B-Raf, in addition to its action on RET, VEGFR, PDGF, and c-Kit. These patients have maintained their response to sorafenib for longer than 10 years. This is remarkable as in our own experience, patients with aggressive metastatic disease exhibited a poor response to sorafenib [33]. Therefore, a question to be raised is whether loss of chromosome 22 could serve as a marker of good response to Raf inhibition therapy.

The finding of a novel in-frame insertion variant *NF1* (p.Thr1065Asnfs*24) in the same patient harboring a germline *NF1* pathogenic variant, strongly suggests biallelic inactivation of this tumor suppressor gene [34, 35]. Neurofibromatosis 1 (NF1) is a multisystem disorder characterized by café-au-lait spots of the skin, intertriginous freckling, multiple cutaneous neurofibromas, subcutaneous or deep nodular neurofibromas, plexiform neurofibromas, and characteristic ocular signs [36]. Many clinical features of NF1 increase in frequency with age, and some individuals who have unequivocal NF1 as adults cannot be diagnosed in early childhood, before NF1 features become apparent [37]. MTC has been associated with NF1 but up to recent reports, it was unclear whether these tumors were caused by a second hit of the *NF1* gene. There are few reports demonstrating association of MTC with NF1, and while most of these reports did not include germline or somatic genetic analysis, at least 2 studies identified both *RET* and *NF1* germline variants in the same patients [38-41].

More recently, Shi et al, identified a pathogenic somatic *NF1* variant in a MTC tumor of a patient with the diagnosis of NF1 [23]. After this report, the analysis of the *NF1* gene in 2 patients with nonhereditary, non-RET, and non-RAS MTC identified *NF1* somatic pathogenic variants: 1 in a patient with confirmed diagnosis of NF1 and another in a non-NF1 patient with 2 acquired somatic *NF1* genetic alterations [22]. These findings support a pathogenic role of *NF1* in MTC tumorigenesis. The loss of neurofibromin function, a protein known to downregulate the Ras pathway, can activate the GTPase activity of Ras/MAPK and PI3K/AKT/mTOR pathways [36], potentially explaining its role as a driver in MTC.

Previous whole-exome studies on sporadic MTC have been limited by a lack of data regarding the correlation between tumoral pathogenic variants and clinical and pathological features. Our study offers valuable insights to the literature, shedding light on the molecular profile of sporadic MTC in a well-characterized cohort of patients with aggressive and early-onset disease. These molecular findings, along with patient's clinical data, may hold potential clinical implications for tailoring therapies and optimizing patient management.

In the current landscape of potent RET-specific inhibitors, the demonstration of the high frequency of *RET* mutations, along with the importance of the Ras–MAPK pathway in tumor development and growth (93.3% of the tumors analyzed had driver variants observed in this pathway), solidifies the importance of tumor somatic analysis to identify the best therapeutic modality for young patients with advanced disease. Furthermore, our findings suggest a potential role of SCNAs as a marker of response to therapy.

We acknowledge the limitations of our study, particularly the relatively small sample size, despite its inclusion of a particularly unique, rare, and uncommon cohort. The utilization of both primary tumor and lymph node samples, especially in cases where primary tumor samples were unavailable, alongside the extensive use of formalin-fixed paraffin-embedded tissue samples, can be attributed to the retrospective nature of our study. Furthermore, it is important to recognize the constraints of exomic sequencing analysis, particularly in its ability to analyze rearrangements and its limitation in examining regulatory and deep intronic regions compared with genome sequencing.

Despite these limitations, we believe our study makes a significant contribution, particularly as it is the first of its kind focusing solely on young patients with aggressive disease. Given our decision to specifically select a cohort resembling our patients with MEN2B—comprising young individuals with aggressive disease—the sample size was inherently limited for analyzing correlations between molecular abnormalities and disease aggressiveness behavior.

This study confirms *RET* as a major genetic driver (80%) even in young patients with nonhereditary MTC and demonstrates *NF1* as a new driver in addition to *RAS* in *RET*-negative tumors. The finding of a germline and somatic *NF1* variant in a young patient without a previous diagnosis or family history of NF1 should serve as an alert for a thorough evaluation to exclude NF1. In our cohort, SCNAs were prevalent and mostly found in *RET*-positive tumors. In this limited cohort of patients with aggressive disease, we failed to demonstrate a correlation with a poor outcome but identified a potential role of loss of chromosome 22 as a marker of TKI treatment response. The clinical significance of these findings is still unclear but deserves further investigation.

## Data Availability

Original data generated and analyzed during this study are included in this published article or in the data repositories listed in References.

## Abbreviations

CEA: carcinoembryonic antigen
LOH: loss of heterozygosity
MEN: multiple endocrine neoplasia
MTC: medullary thyroid carcinoma
*NF1*: neurofibromin 1 gene
NF1: neurofibromatosis type 1
SNCA: somatic copy number alteration
TKI: tyrosine kinase inhibitor
VUS: variants of unknown significance
